# Flow pumping by external periodic shear applied to a soft interface

**DOI:** 10.1038/s41598-021-94398-9

**Published:** 2021-07-22

**Authors:** Shima Nezamipour, Ali Najafi

**Affiliations:** 1 Department of Physics, Institute for Advanced Studies in Basic Sciences (IASBS), Zanjan, 45137-66731 Iran; 2 Research Center for Basic Sciences and Modern Technologies (RBST), Institute for Advanced Studies in Basic Sciences, Zanjan, Iran

**Keywords:** Fluid dynamics, Design, synthesis and processing

## Abstract

Flow pumping in viscous fluids is of prime importance in micro-fluidic applications. Here we show that a single colloidal particle in front of a soft wall, manipulated by external means like an optical tweezer, can pump the ambient viscous fluid. The particle, moving back and forth parallel to the soft wall, can produce an averaged net flow in a direction perpendicular to the wall. Using a perturbative scheme, we present the results. Analysis show that this flow in terms of capillary number, scales as ${\text {Ca}}^2$.

Studying the propagation of flow near boundaries and walls of channels, has attracted an increasing interest recent years. The interests in such systems include, flow pumping, flow control and flow mixing in micro-fluidic applications^1–4^. In such systems, different mechanisms have been proposed for manipulating particles^5^ or pumping fluids using for example, acoustic and optical means^6, 7^.

The most important challenge in this field, is the domination of viscosity over inertia in low Reynolds condition, a regime that can be well realized in micrometer scales. The well known Scallop theorem in low Reynolds conditions, sets important constraints on the methods by which one can pump the flow by periodic actuation of some internal structure of the system^8, 9^. For example, a colloidal particle trapped by an optical tweezer which has enforced the particle to move in a periodic orbit, is not able to produce net flow. This is valid for either cases of one dimensional back and forth motion or motion along a circular orbit. In low Reynolds condition, the flow is reversible and, always the flow produced in the second half-period will cancel the flow of the first half-period and, on average the flow will vanish. A flow pump, must have an internal structure with special design to work successfully^10^. In practice, this condition can be achieved by at least, two colloidal particles moving near a rigid wall or three colloidal particles moving far from rigid walls.

An alternative way is to use a single colloidal particle moving near a soft wall. In this article, our goal is to show that, a single colloidal particle under the control of an optical tweezer, can produce a net flow near a soft wall. This particle playing a simple back and forth motion parallel to the wall, will produce an average net flow in direction perpendicular to the soft wall. Soft interface is the main element that makes it possible to induce net flow with a single particle. This is not in contradiction with the scallop theorem. Actually, the deformation modes of the interface can be considered as extra degrees of freedom that can play the role of additional particles.

Deformable boundaries could be realized with either real surfaces or physical interfaces between different fluids that are in contact. Various elastic modulus can capture the deformability, but a simple and frequently addressed moduli that we want to concentrate is the surface tension. A very vast range of surface tension from ${\text {mN}}/{\text {m}}$ to $\mu {\text {N}}/{\text {m}}$ can be realized in experiments^11^.

Nonlinearity associated with the deformability of wall, makes it hard to find analytic solutions to the flow problems near a soft wall. However, using perturbative methods it is possible to use the results of flow near flat wall and find partial solutions to the flow confined by a deformable soft wall.

Investigations corresponding to flow patterns near walls, has a long history. One of the early investigations that considered the influence of a soft wall on a particle motion dates back to the early work of Aderogba and Blake who have introduced the Green’s functions of a point force in the vicinity of a fluid-fluid interface^12^. They have assumed that the surface tension is very high which holds the interface flat. Considering a similar assumption, the drag force and the hydrodynamic torque acting on a finite size sphere near the interface has been obtained by Lee and coworkers^13^. The results obtained by Lee and coworkers have been investigated experimentally with optical tweezer^14^. The hydrodynamic nonlinearity due to the flexibility of the membranes has interesting effects^15^. As recently observed, a small particle sedimenting near an elastic membrane is repelled from the membrane and its normal velocity has a nonlinear dependency to the sedimenting force^16, 17^. This hydrodynamic lift force has been analytically studied^18, 19^. This normal force (lift force) breaks the time-reversal symmetry of Stokes flow. Inspiring this interesting result, Trouilloud *et. al.*, theoretically predicted that a micro-swimmer performing reciprocal actuations is able to move near a soft interface^20^. In agreement with this prediction, an analytical and numerical study on the dynamics of a time-reversible squirmer illustrated that it is able to move near a weakly deformable interface^21^.

**Figure 1:**
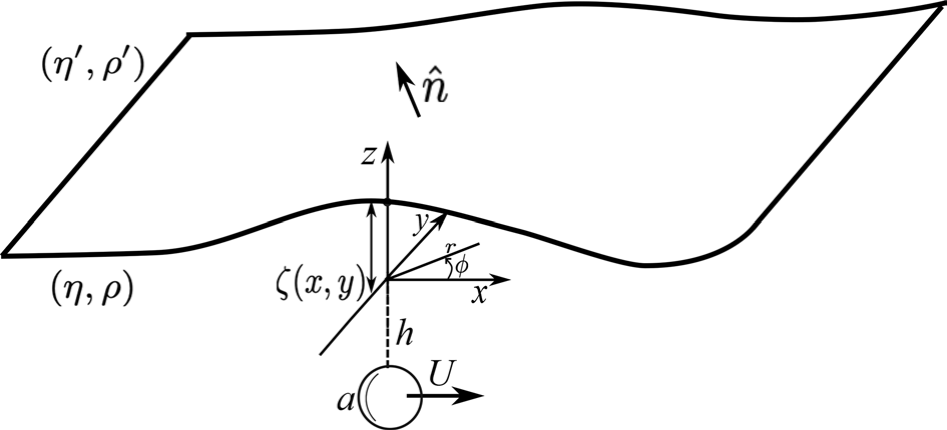
A soft interface separates two immiscible fluids and the shear exerted on this interface from a moving micro-particle, will deform it. The deformation pattern shown here, is pictorial and it does not necessarily shows a real pattern for a specific motion of a micro-particle.

The rest of this article is organized in the following way. In “Model” section, we first introduce the model that we want to study. Then in “Governing equations” and “Perturbation analysis” sections, we present the governing equations and the details of perturbation scheme that we use to solve the equations. In “Steady state results” section, we present the flow pattern and hydrodynamic force distribution for an auxiliary steady state problem that will be used to obtain the solutions to our real problem. Numerical and scaling results for the pumping flow will be presented in “Average pumping flow ” and “Dimensional analysis” sections, respectively.

## Model

As shown in Fig. 1, consider a spherical micro-particle with radius *a* that is moving near a soft and initially flat wall. This flexible wall separates two immiscible fluid phases which are characterized by their densities and viscosities given by $(\eta ,\rho )$ and $(\eta ',\rho ')$, respectively. The sphere is located in the phase that is denoted by unprimed variables. The soft wall can either be a simple interface or a physical membrane with respective elastic modulus. Here, for simplicity, we consider a soft interface with an isotropic surface tension denoted by $\gamma$. With the setup that is introduced here, we aim to consider the hydrodynamic flow pattern near an interface that is under a tangential periodic shear force. The micro-particle that is trapped by an optical tweezer, is the source of this shear force. As an approximation that can further simplify the problem, it is assumed that the rotational motion of the particle is balanced by a suitable external torque. Along this task, we let the trapped particle to move in a prescribed 1-dimensional periodic trajectory parallel to the interface.

To parametrize the geometry, as shown in Fig. 1, we choose a laboratory reference frame where the position of the interface, in its undeformed state, is given by $z=0$ plane. As shown in the picture, both cylindrical variables $(r, \phi , z)$ and Cartesian variables (*x*, *y*, *z*) will be used to parametrize the problem. In this coordinate system and in terms of Cartesian variables, the position of the micro-particle is given by:

1 $$\begin{aligned} {\mathbf{x}} _{\text {p}}(t)=\left( x_{\text {p}}\sin (\omega t),0,-h\right) , \end{aligned}$$

where *h* denotes the distance between sphere and interface, $x_0$ stands for the amplitude of motion and $\omega$ shows the frequency of the motion. The instantaneous velocity of the sphere is given by ${\mathbf{U}} _{\text {p}}(t)=U_{\text {p}} \cos (\omega t) \hat{x}$ with $U_{\text {p}}=\omega x_{\text {p}}$. As a result of the shear forces exerted from moving sphere, the interface will be deformed. In the laboratory frame, time dependent deformation pattern of the interface can be denote by $z=\zeta (x,y,t)$.

In addition to the flow pattern that is generated in the fluid, the deformation profile $z=\zeta (x,y,t)$ is among the unknown variables that we aim to determine. In the following part we will present the necessary dynamical equations that will help us to calculate the unknown variables.

## Governing equations

To investigate the dynamics of this system, we denote by ${\mathbf{u}}({\mathbf{x}})$ and $p({\mathbf{x}})$, velocity and pressure fields in the fluid medium. To distinguish the field variables at both sides of the interface, we will use the primed and unprimed symbols for top and bottom fluids, respectively.

To write the dynamical equations, we benefit the approximations that are essential for our micron-scale set up. Denoting the dimensionless Reynolds number by ${\text {Re}}={\rho U_{\text {p}} a}/{\eta }$, we will assume that ${\text {Re}}\ll 1$. This will restrict the dynamics of the fluid to the viscosity dominated regime. In this limit, the Stokes and continuity equations will determine the flow filed. In terms of the dynamical variables, these equations read as:

2 $$\begin{aligned} \eta \nabla ^2{\mathbf{u}} -\nabla p ={0},~~~~\nabla \cdot {\mathbf{u}} ={0}. \end{aligned}$$

Similar equations will be held for primed variables.

It should be noted that in general, the low Reynolds condition does not guarantee the steadiness of the flow. In our system for ${\text {Re}}\ll 1$, the flow pattern is assumed to reach an instantaneous steady state. As a result of this approximation, time does not appear in the Stokes equation explicitly. However as the sphere moves slowly in the medium, the flow will have a quasi-steady time evolution.

The quasi-steady approximation will allow us to consider a true steady state problem from which we can extract the results for our quasi-steady state problem. Let us define the steady state (instantaneous) problem with a micro-particle that is located at ${\mathbf{x}} _{\text {p}}^{\text {s}}=(0,0,-h)$ with its surface velocity given by ${\mathbf{U}} _{\text {p}}=U_{\text {p}}{\hat{x}}$. We denote the deformation pattern and velocity field of this problem by $\zeta ^{\text {s}}(x,y)$ and ${\mathbf{u}}^{\text {s}}(x,y,z)$, respectively. The solution to our real quasi-steady state problem at an arbitrary time *t* and in laboratory frame, can be written as:

3 $$\begin{aligned} \zeta (x,y,t)=\zeta ^{\text {s}}(x-x_{\text {p}}(t),y),~~~{\mathbf{u}}(x,y,z,t)={\mathbf{u}}^{\text {s}}(x-x_{\text {p}}(t),y,z), \end{aligned}$$

where, in the steady state solutions we have replaced the position and velocity of the particle by: ${\mathbf{U}} _{\text {p}}={\mathbf{U}} _{\text {p}}(t)$ and $x_{\text {p}}(t)=\int _0^{t}U_{\text {p}}(t')dt'$, respectively.

### Boundary conditions

Boundary conditions for the above mentioned steady state problem, are given by:

4 $$\begin{aligned} {\mathbf{u}} ^{\text {s}}({\mathbf{x}} |_{\text {{on~sphere}}} )={\mathbf{U}} _{\text {p}},~~~ {\mathbf{u}} ^{\text {s}}(|{\mathbf{x}} |\rightarrow \infty )={\mathbf{0}} ,~~~{\mathbf{u}}^{\text {s}}(x,y,z=\zeta ^{\text {s}})={\mathbf{u}}'^{\text {s}}(x,y,z=\zeta ^{\text {s}}), \end{aligned}$$

where the last equation represents the continuity of velocity field at the position of interface.

Regarding the energy stored in the interface, both the tension and gravitational energy, the components of the stress tensor, obey the following boundary conditions at the position of interface^16^:

5 $$\begin{aligned} {\hat{t}}\cdot ({\mathbf{T}} '^{\text {s}}-{\mathbf{T}} ^{\text {s}})\cdot {\hat{n}}=0,~~~ {\hat{n}}\cdot ({\mathbf{T}} '^{\text {s}}-{\mathbf{T}} ^{\text {s}})\cdot {\hat{n}} -\gamma (\nabla \cdot {\hat{n}})-g\Delta \rho \zeta ^{\text {s}} =0, \end{aligned}$$

where ${\hat{n}}$ and ${\hat{t}}$ denote two unit vectors that are locally normal and tangent to the interface and $(1/2)\nabla \cdot {\hat{n}}$ denotes the mean curvature of the interface. Density contrast is denoted by $\Delta \rho =\left( \rho -\rho '\right)$. We note that in terms of the interface shape function $H=z-\zeta ^{\text {s}}$, the normal vector can be written as: ${\hat{n}}=\nabla H/|\nabla H|$. Discontinuity in the normal stress that is reflected in the last part of the above equation, is the main mechanism that can initiate deformation in the membrane. Regarding the detail structure of this equation, three different forces namely, viscous, tension and gravity, compete in force balance. Relative strength of these forces can be given in terms of two dimensionless numbers:

6 $$\begin{aligned}&\alpha =\frac{\text {gravitational force}}{\text {viscous force}}=\frac{g a^{2}\Delta \rho }{\eta U_{\text {p}}},\nonumber \\&\beta =\frac{\text {surface~tension}}{\text {viscous tension}}=\frac{\gamma }{\eta U_{\text {p}}}, \end{aligned}$$

where, $U_{\text {p}}$ is a velocity scale that was defined before. Including the ratio between viscosities, $\lambda ={\eta '}/{\eta }$, these three dimensionless numbers can be used to determine the physical state of the system under investigation. For very small viscous tension, $\alpha ^{-1}\ll 1$ and $\beta ^{-1}\ll 1$, hence the membrane remains flat. We will use this observation in developing a perturbative procedure to solve the dynamical equations. It is worth mentioning that a new length scale, namely the capillary length can be defined as: $\ell _{\text {c}}=\sqrt{\gamma /g\Delta \rho }$. At scales much larger than this capillary length, the gravity dominates over the surface tension. Capillary length is a relevant length scale when the gravity and surface tension have the same order of magnitudes. In other regime where the effects of gravity are negligible, a dimensionless number so called the capillary number ${\text {Ca}}=\beta ^{-1}$ plays the role of a control parameter instead of capillary length. We present our main results in the latter regime.

To finish with the formulation, we need an equation that couples the dynamics of the interface and the fluid. We note that in the laboratory frame and as a result of no-slip boundary condition on the wall, the trajectory of a particular point of the interface should follow exactly the path of the fluid particles that are locally in contact with points on the wall. In terms of the fields in laboratory frame, we have ${\hat{n}}\cdot {\mathbf{u}}(x,y,z=\zeta )=\partial _t\zeta /\sqrt{1+\nabla \zeta \cdot \nabla \zeta }$, where $\partial _t=\partial /\partial t$. In terms of the fields corresponding to the previously defined steady state problem, we will have:

7 $$\begin{aligned} {\hat{n}}\cdot {\mathbf{u}}^{\text {s}}(x,y,z=\zeta ^{\text {s}})=-U_{\text {p}}(\partial _x\zeta ^{\text {s}})/\sqrt{1+\nabla \zeta ^{\text {s}}\cdot \nabla \zeta ^{\text {s}}}. \end{aligned}$$

The set of boundary Eqs. (4), (5) and (7), will allow us to find complete information about the shape of interface and the velocity fields on both sides of the interface.

## Perturbation analysis

Even though the governing equations at ${\text {Re}}=0$ are linear in terms of velocity field, the boundary conditions at the position of interface are highly nonlinear with respect to the deformation profile, $\zeta ^{\text {s}}(x,y)$. This nonlinearity in terms of $\zeta ^{\text {s}}$, will result velocity profiles at both sides of the interface that are nonlinear in terms of the sphere velocity $U_{\text {p}}$. Our strategy to overcome this nonlinearity, is to use some approximations. It is obvious that for either cases of a system with hard interface ($\beta ^{-1}={\text {Ca}}\ll 1$) or a system with large density contrast or large gravity ($\alpha ^{-1}\ll 1$), we expect a very small deformation pattern. Regarding this observation, we can define a small parameter denoted by $\epsilon =({\alpha +\beta })^{-1}$ and set up a perturbative expansion of the required fields in terms of this small dimensionless quantity. Formal expansion of all fields read as:

8 $$\begin{aligned} \{\mathbf{u }^{\text {s}},p^{\text {s}},T^{\text {s}},...\}= & {} \{\mathbf{u }^{\text {s}}_{0},p^{\text {s}}_{0},T^{\text {s}}_{0},...\} +\epsilon \{\mathbf{u }^{\text {s}}_{1},p^{\text {s}}_{1},T^{\text {s}}_{1},...\}+{\mathscr {O}}(\epsilon ^2),\nonumber \\ \zeta ^{\text {s}}(x,y)= & {} \epsilon \zeta ^{\text {s}}_{1}(x,y)+\epsilon ^{2} \zeta ^{\text {s}}_{2}(x,y)+{\mathscr {O}}(\epsilon ^3). \end{aligned}$$

As it is expected, at zeroth order of $\epsilon$, the interface remains flat and the equations are linear. To be mathematically precise, one should note that the above perturbative expansion should be written for dimensionless variables. Using characteristic length and time scales given by *a* and $(a/U_{\text {p}})$ and denoting the scales for pressure by $\eta U_{\text {p}}/a$ and $\eta 'U_{\text {p}}/a$, we can transform the equations to dimensionless form. Following such transformation, small quantity denoted by $\epsilon$, will arise naturally. However, to keep the notation as minimal as possible, we prefer to proceed with the dimensional form of the equations.

The above perturbation analysis can work in all regimes but, as we stressed before, we will mainly concentrate on the regime where the capillary number ${\text {Ca}}$ plays the main role. In this regime $\beta \gg \alpha$ and $\epsilon \sim {\text {Ca}}$. In the following parts, we will present the equations and results up to the leading orders of $\epsilon$.

### Zero order equations

The equations for the zeroth order fields are given by:

9 $$\begin{aligned} \eta \nabla ^2{\mathbf{u}} ^{\text {s}}_{0}-\nabla p^{\text {s}}_{0} =0,~~~\nabla \cdot {\mathbf{u}} ^{\text {s}}_{0}=0, \end{aligned}$$

with similar equations for primed fields. The flow fields are subjected to the following boundary conditions:

10 $$\begin{aligned}&{\mathbf{u}}^{\text {s}}_{0}({\mathbf{x}} |_{\text {on sphere}} )={\mathbf{U}} _{\text {p}},~~~ {\mathbf{u}} ^{\text {s}}_{0}(|{\mathbf{x}} |\rightarrow \infty )=0,~~~{u}^{'\text {s}}_{0z}(x,y,z=0)={u}^{\text {s}}_{0z}(x,y,z=0)=0,\nonumber \\&{u}^{'\text {s}}_{0l}(x,y,z=0)={u}^{\text {s}}_{0l}(x,y,z=0),~~~ {T}^{'\text {s}}_{0lz}(x,y,z=0)={T}^{\text {s}}_{0lz}(x,y,z=0), \end{aligned}$$

where *l* can be either *x* or *y*.

### First order equations

Having in hand the zeroth order results for the flow fields, we can calculate the first order deformation by solving the following differential equation:

11 $$\begin{aligned} \left( 1-\ell _{\text {c}}^2 \nabla ^{2} \right) \epsilon \zeta ^{\text {s}}_{1}(x,y)= \frac{1}{g\Delta \rho }({T}'_{0zz}-{T}_{0zz}). \end{aligned}$$

After calculating the interface shape at the first order, we will be able to obtain the first order correction to the flow profile. At ${\mathscr {O}}(\epsilon )$, equations for the flow fields are given by:

12 $$\begin{aligned} \eta \nabla ^2{\mathbf{u}} ^{\text {s}}_{1}-\nabla p^{\text {s}}_{1} =0,~~~\nabla \cdot {\mathbf{u}} ^{\text {s}}_{1}=0, \end{aligned}$$

and corresponding boundary conditions are given by:

13 $$\begin{aligned}&{\mathbf {u}} ^{\text {s}}_{1}({\mathbf {x}} |_{\text {on sphere}} )=0,\,\,\,{\mathbf{u}} ^{\text {s}}_{1}(|{\mathbf{x}} |\rightarrow \infty )=0,\nonumber \\&\Delta {\mathbf{u}}^{\text {s}}_1={\mathbf{u}}^{\text {s}}_{1}(x,y,z=0)-{\mathbf{u^\prime}}^{\text {s}}_{1}(x,y,z=0)=-\zeta ^{\text {s}}_1 \partial _z[{\mathbf{u}}^{\text {s}}_{0}(x,y,z=0)-{\mathbf{u}}^{\prime{\text {s}}}_{0}(x,y,z=0) ],\nonumber \\&u^{\text {s}}_{1z}(x,y,0)=-U_{\text {p}}\partial _x\zeta ^{\text {s}}_1-\zeta ^{\text {s}}_1\partial _zu^{\text {s}}_{0z}+{\mathbf{u}}_{0}^{\text {s}}\cdot \nabla \zeta _{1}^{\text {s}},\nonumber \\&\Delta {T}^{\text {s}}_{1zt}={\hat{z}}\cdot ({\mathbf{T}}^{\prime {s}}_{1}-{\mathbf{T}} ^s_{1})\cdot \hat{t}=-\zeta ^s_{1}{\hat{z}}\cdot \partial _z({\mathbf{T^\prime}}^s_{0}- {\mathbf{T}} ^s_{0})\cdot \hat{t}+\nabla \zeta ^s_{1}\cdot ({\mathbf{T}}^{\prime s}_{0}-{\mathbf{T}} ^s_{0}) \cdot \hat{t}\nonumber \\&+A \nabla ^{2} \zeta ^s_{1}\nabla \zeta ^s_{1} \cdot \hat{t} -B \zeta ^s_{1}\nabla \zeta ^s_{1}\cdot \hat{t}, \end{aligned}$$

where $$A={\eta U_{\text {p}}}{}\frac{\ell _{\text {c}}^2}{\ell _{\text {c}}^2+a^2}$$ and $$B=A/\ell _{\text {c}}^2$$.

## Steady state results

Having introduced the steady state equations for zeroth and first order fields, we can present their solutions. Our final goal in this part is to obtain the first order flow and the first order hydrodynamic force acting on the micro-particle.

**Figure 2:**
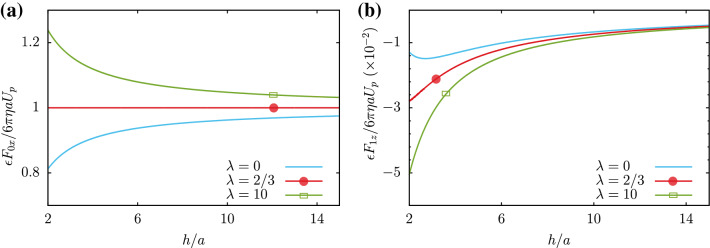
(**a**) The zeroth order hydrodynamic force acting on the particle in terms of the distance to wall is plotted. This force is parallel to the wall. (**b**) The first order force is perpendicular to the wall and it is plotted in terms of the distance to wall. Parameters are ${\mathbf{U}}_{\text {p}}=U_{\text {p}}{\hat{x}}$ with ${U}_{\text {p}}>0$ and ${\text {Ca}}=0.1$.

### Zeroth order results

So far, we have introduced the perturbative equations for the steady state problem. As it is reflected in Eqs. (9) and (10), the zero order flow corresponds to the flow due to a moving sphere near a flat wall with vanishing normal velocity and continuous tangential velocity on the wall. This is a classical fluid dynamic problem with a general solution that can be obtained by using the Lorentz reciprocal theorem^22^. Using such method, one can obtain results that take into account the finite radius of the sphere. However, here and to avoid the complicated mathematical structure of the results, we restrict ourselves to a limiting case of a very small sphere and expand all the results in powers of small parameter (*a*/*h*). At the leading order of (*a*/*h*), the velocity field can be presented in terms of the flow corresponding to a point force ${\mathbf{f}}_0=6\pi \eta aU_{\text {p}}$. In terms of the Green’s function, the velocity can be written as:^12^ $u^{\text {s}}_{0i}=\frac{1}{8 \pi \eta } \sum _{j}G_{ij}f_{0j}$ where,

14 $$\begin{aligned} G_{ij}({\mathbf{x}} ,{\mathbf{x}} _p)= & {} \frac{\delta _{i,j}}{r}+\frac{r_i r_j}{r^3}+\left( \frac{(1-\lambda ) \delta _{j,\alpha } \delta _{\alpha ,k}}{\lambda +1}-\delta _{j,3} \delta _{3,k}\right) \left( \frac{\delta _{i,k}}{R}+\frac{R_i R_k}{R^3}\right) \nonumber \\&\quad +\frac{2 h \lambda }{\lambda +1} \left( \delta _{j,\alpha } \delta _{\alpha ,k}-\delta _{j,3} \delta _{3,k}\right) \left( \frac{\delta _{i,k} (h+R_3)-\delta _{i,3} R_k+\delta _{3,k} R_i}{R^3}-\frac{3 (h+R_3) R_i R_k}{R^5}\right) , \end{aligned}$$

where ${\mathbf{r}} =(x,y,z+h)$, ${\mathbf{R}} =(x,y,z-h)$, and *i*, *j*, *k* can take values of 1, 2, 3 but $\alpha$ is restricted to 1 or 2. Using the Lorentz reciprocal theorem, the hydrodynamic force acting on the sphere, can be written as:^22^

15 $$\begin{aligned} {\mathbf{F}^{\text {s}}_{0}}={-}6\pi \eta aU_{\text {p}} \Gamma _0 {\hat{x}},~~~\Gamma _0=\left( 1-\frac{3}{16}\frac{2-3\lambda }{1+\lambda }\frac{a}{h}\right) +{\mathscr{O}}\left( \frac{a}{h}\right) ^2. \end{aligned}$$

In terms of (*h*/*a*), this drag is plotted in Fig. 2a. As one can see, for $\lambda =\eta '/\eta \ge 2/3$, increasing the distance from wall will decrease the drag until it reaches to its asymptotic value, corresponding to the drag in a homogenous single-phase medium.

Note that the case with $\lambda =1$ does not correspond to a single fluid system. To see the reason, we note that for the zeroth order problem, distribution of normal stress over the wall does not vanish in the limit of $\lambda =1$. This normal stress at the zeroth order, is the driving force that will deform the wall.

Normal stress distribution over the interface is another important quantity that we can calculate. The leading order normal stress difference at small (*a*/*h*), can be written as^22^:

16 $$\begin{aligned} ({T}'_{0zz}-{T}_{0zz})= \frac{\eta U_{\text {p}}}{a}f(r,\phi )= \frac{\eta U_{\text {p}}}{a}R(r)\cos \phi , \end{aligned}$$

where $R(r)=\Gamma _0\frac{9h^2ra^2}{(h^2+r^2)^{5/2}}$. In the following part we will use this result to obtain the deformation pattern of the membrane.

### First order deformation

After calculating the lowest order velocity profile and subsequently obtaining the lowest order stress tensor, we will be able to study the first order correction to the deformation pattern. According to Eq. (11), the discontinuity of the stress tensor (its normal component) can be used to calculate the deformation pattern. Using Eq. (11), the deformation pattern in Fourier space can be written as:

17 $$\begin{aligned} \epsilon \tilde{\zeta }_1({\mathbf{q}})=a{\text {Ca}}(\ell _{\text {c}}/a)^ 2(1+\ell _{\text {c}}^2 q^2)^{-1} \times \int d^2{\mathbf{r}}e^{i{\mathbf{q}}\cdot {\mathbf{r}}}f({r},\phi ). \end{aligned}$$

Integrating over wave vector, we will arrive at the following result for the deformation field:

18 $$\begin{aligned} \epsilon \zeta ^{\text {s}}_1(r,\phi )=a{\text {Ca}}(\ell _{\text {c}}/a)^2g(r)\cos \phi ,~~~g(r)=\int _0^{\infty }r'dr'\int _0^{\infty }qdq\frac{J_0(qr)J_0(qr')R({r'})}{1+(\ell _{\text {c}}/a)^2 (aq)^2}. \end{aligned}$$

For a case with very large capillary length ($\ell _{\text {c}}\gg a$ or $\epsilon \sim {\text {Ca}}$), the shape of the interface can be achieved as:

19 $$\begin{aligned} \epsilon \zeta _{1}^s(r,\phi )=3a{\text {Ca}}\times \frac{h}{r}\left( 1-\frac{3}{16}\frac{2-3 \lambda }{1+\lambda }\frac{a}{h}\right) \left( 1-\frac{h}{(h^2 + r^2 )^{\frac{1}{2}}}\right) \cos \phi . \end{aligned}$$

**Figure 3:**
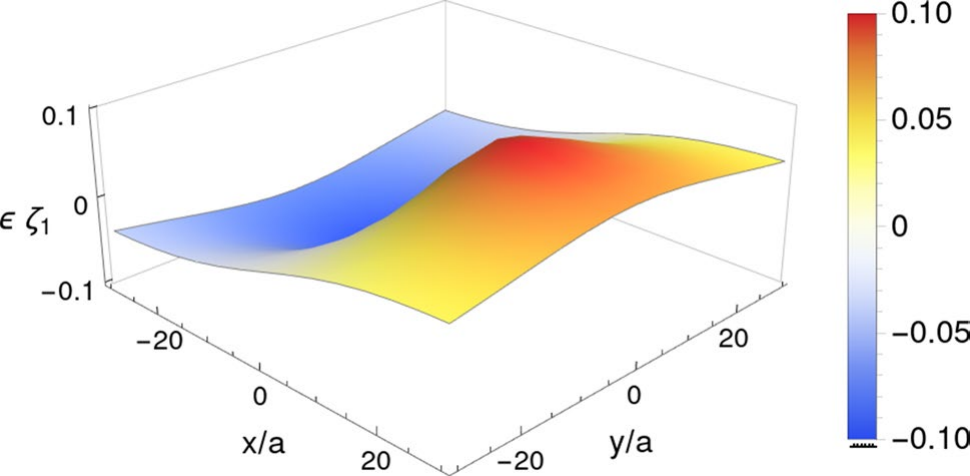
Deformation pattern corresponding to a sphere located at position $(0,0,-10)$ and moving in $+{\hat{x}}$ direction. As it is reflected from this figure, the membrane experiences a sink (rise) in front (back) of the moving sphere. Other parameters are $\lambda =\frac{3}{2}$ and ${\text {Ca}}=0.1$.

Figure 3 shows an example of the interfacial deformation pattern for specific values of the parameters. As it is seen in this figure, the interface experiences a sink (rise) in front (back) of the moving sphere. The front sink observed in the interface, is due to the positive pressure produced by a moving sphere in its front.

### First order flow

As we showed before, the zeroth order results do not take into account the deformation of the interface. Our main interest in this article is the first order correction to the velocity profile that takes into account the flexibility of the membrane. As we will see later, this contribution will give a net flow for a periodic and reciprocal motion of the sphere when we average it over time.

**Figure 4:**
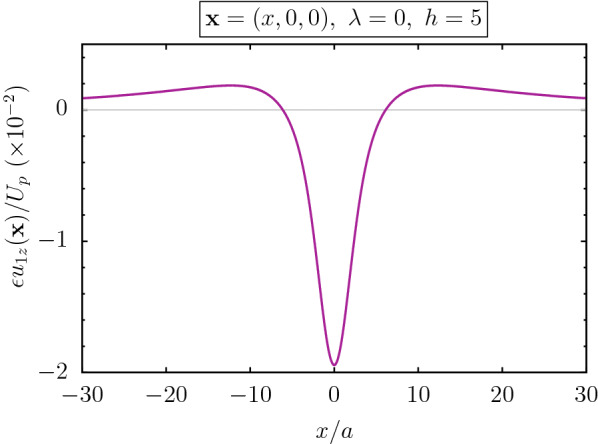
Boundary velocity $u_{1z}^{\text {s}}(x,y=0,z=0)$ is shown. Parameters are $\lambda =0$, $h/a=5$, ${\text {Ca}}=0.1$.

Eqs. (12) and (13) show that the first order problem is a stokes flow with vanishing body force that is subjected to an inlet normal velocity profile given at the position of undeformed interface ($z=0$). Moreover, for this flow, the tangential velocity and the components of the stress tensor are discontinuous at $z=0$ and the velocity must vanish on the surface of the sphere. Having in hand the zeroth order flow and first order deformation pattern, the boundary normal velocity can be obtained. As a function of $\rho$ and $\phi$ in the cylindrical coordinate system, this boundary velocity has the following form:

20 $$\begin{aligned} & \epsilon u_{1z}^{\text{s}}(\rho ,\phi ,0) = -3 Ca U_p a\left( \frac{ h^{2}}{2 \rho _{0}^{3}}+\left( \frac{ h^{2} \left( 2 h^{2}+3 \rho ^{2}\right) }{2 \rho ^{2} \rho _{0}^{3}}-\frac{ h}{\rho ^{2}}\right) \cos (2 \phi )\right) +{\mathscr {O}}\left( \frac{a}{h}\right) ^{2}, \end{aligned}$$

where $\rho _0=\sqrt{\rho ^2+h^2}$. Profile of this inlet normal velocity, is plotted in Fig. 4. As it is reflected from the this result, the inlet velocity points toward $-{\hat{z}}$ direction, that tends to push and repel the particle from wall.

Now, the Lorentz reciprocal relation can help us to find the first order velocity and stress tensor that are denoted by ${\mathbf{u}}^{\text {s}}_1$ and ${\mathbf{T}}^{\text {s}}_1$, respectively. Let us consider a complementary problem with velocity and stress given by ${\mathbf{v}}$ and $\Sigma$, respectively. The Lorentz relation reads as:

21 $$\begin{aligned} (\nabla \cdot \Sigma )\cdot {\mathbf{u}}^{\text {s}}_1 - (\nabla \cdot {\mathbf{T}}^{\text {s}}_1)\cdot {\mathbf{v}}=\nabla \cdot \left( \Sigma \cdot {\mathbf{u}}^{\text {s}}_1- {\mathbf{T}}^{\text {s}}_1\cdot {\mathbf{v}}\right) , \end{aligned}$$

where, short-hand notation for matrix product is used as: ${\mathbf{a}}\cdot \Gamma \cdot {\mathbf{b}}=\sum _{ij}a_i\Gamma _{ij}b_j$. Note that, as before, primed variables are reserved for all fields at the upper fluid (on top of the membrane).

**Figure 5:**
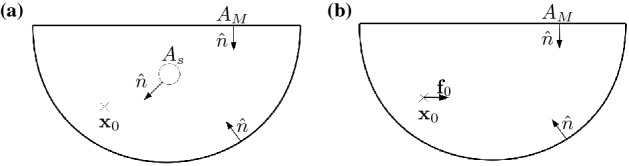
The boundaries of our main problem and the auxiliary problem are shown in parts (**a**) and (**b**), respectively. Observation point ${\mathbf{x}}_0$, shows the point where we are interested to calculate the velocity field.

We aim to calculate the velocity field at an arbitrary observation point ${\mathbf{x}}_0$, located in the lower fluid. We consider a complementary flow that corresponds to a point force located at the observation point ${\mathbf{x}}_0$. This complementary problem is subjected to free-slip velocity at $z=0$ and it vanishes at infinity. For this complementary problem, we have $\nabla \cdot \Sigma =-{\mathbf{f}}_0\delta ({\mathbf{x}}-{\mathbf{x}}_0)$, where ${\mathbf{f}}_0$ is a given force strength exerted on the fluid. One should note that our main problem (first order fields) and the complementary problem are subjected to different boundaries as shown in Fig. 5.

To be more precise, the velocity fields of our complementary problem in upper and lower fluid, ${\mathbf{v}}$ and ${\mathbf{v}}'$, satisfy the following conditions:

22 $$\begin{aligned}&{\mathbf{v}} (|{\mathbf{x}} |\rightarrow \infty )=0,~~~{v^\prime}_{z}(x,y,z=0)={v}_{z}(x,y,z=0)=0,\nonumber \\&{v^\prime}_{l}(x,y,z=0)={v}_{l}(x,y,z=0),~~~ {\Sigma^\prime}_{lz}(x,y,z=0)={\Sigma }_{lz}(x,y,z=0), \end{aligned}$$

where *l* can be either *x* or *y*. Exact solution to this complementary problem, using the well known image method, is presented in^12^. Note that this velocity profile corresponding to a point force was presented before in Eq. (14).

We integrate the Lorentz relation, Eq. (21), over the domain of the lower fluid as shown in Fig. 5a and use the divergence theorem to reach the following relation:

23 $$\begin{aligned} {\mathbf{u}} ^{\text {s}}_1({\mathbf{x}} _0)\cdot {\mathbf{f}} _0= -\int _{A_{M}} {\mathbf{u}}^{\text {s}}_1\cdot \Sigma \cdot {\hat{z}} d^{2}{\mathbf{x}} + \int _{A_M} {\mathbf{v}} \cdot {\mathbf{T}}^{\text {s}}_1\cdot {\hat{z}} d^{2}{\mathbf{x}} -\int _{A_s}{} {\mathbf{v}} \cdot {\mathbf{T}}^{\text {s}}_1\cdot {\hat{n}} d^{2}{\mathbf{x}} , \end{aligned}$$

where we have used the relevant boundary conditions and also benefited the fact that for our main problem $\nabla \cdot {\mathbf{T}}_{1}^{\text {s}}=0$ (no body force exerted). Following a similar procedure and integrating the Lorentz relation over the domain of upper fluid, we will arrive at:

24 $$\begin{aligned} 0 = -\int _{A_M}{} {\mathbf{u'}}_{1}^{\text {s}}\cdot \Sigma '\cdot {\hat{z}} d^2{\mathbf{x}} +\int _{A_M}{} {\mathbf{v'}}\cdot {\mathbf{T'}}_{1}^{\text {s}}\cdot {\hat{z}} d^2 {\mathbf{x}} \end{aligned}$$

Combining Eqs. (24) and (23), we will have:

25 $$\begin{aligned} {\mathbf{u}} ^{\text {s}}_1({\mathbf{x}} _0)\cdot {\mathbf{f}} _0= & {} \int _{A_{M}} \Delta {\mathbf{u}}^{\text {s}}_1 \cdot \Sigma '\cdot {\hat{z}} d^2{\mathbf{x}} + \int _{A_{M}} {\mathbf{u}} ^{\mathbf{s}}_1\cdot \Delta \Sigma \cdot {\hat{z}} d^2{\mathbf{x}} \nonumber \\&\quad - \int _{A_M}{} {\mathbf{v}} \cdot \Delta {\mathbf{T}}^{\text {s}}_1\cdot {\hat{z}} d^2{\mathbf{x}} -\int _{A_s}{} {\mathbf{v}} \cdot {\mathbf{T}}^{\text {s}}_1\cdot {\hat{z}} d^2{\mathbf{x}} , \end{aligned}$$

where $\Delta {\mathbf{u}}^{\text {s}}_1$ and $\Delta {\mathbf{T}}^{\text {s}}_1$ have been defined in Eq. (13) and $\Delta \Sigma =\Sigma '-\Sigma$. It should be noted that ${\hat{t}}\cdot \Delta \Sigma \cdot {\hat{z}}=0$ then, in the second integral of the above equation, the *z* component of the velocity, $u^{\text {s}}_{1z}$ will appear, and it is given in Eq. (13). Furthermore, as the normal component of ${\mathbf{v}}$ in the third integral vanishes, then $\Delta {T}^{\text {s}}_{1zt}$ will appear in the equation and it is given in Eq. (13).

Having in hand the exact solution to the complementary flow, partially presented in Eq. (14), we can use the above equation and numerically calculate the velocity profile. Choosing point force in directions parallel and perpendicular to the wall, we can obtain the parallel and perpendicular components of the velocity.

Dealing with the first, second and the third integrals that appeared in the above equation, is straightforward. The main difficulty comes with the fourth integral that needs the distribution of first order tension over the sphere as an input function. In other words, the above equation can be considered as an integral equation for the first order flow pattern. Far from the sphere and as an approximation, we can neglect the effects of this integral and proceed with the first terms.

### First order force

To calculate the first order hydrodynamic force exerted on the sphere, ${\mathbf{F}}^{\text {s}}_{1}$, the Lorentz relation can be used. Here we choose a complementary flow that corresponds to the flue due to a spherical particle translating with velocity ${\mathbf{V}}$ at point ${\mathbf{x}}_{\text {p}}$. This complementary problem satisfies the stokes equation ($\nabla \cdot \Sigma =0$) with following boundary conditions:

26 $$\begin{aligned}&{\mathbf{v}}(|{\mathbf{x}}|\rightarrow \infty )={0},~~{\mathbf{v}}({\mathbf{x}}_{\text {p}} )={\mathbf{V}},~~~ \Sigma^{'}_{lz}(x,y,0)=\Sigma _{lz}(x,y,0)\nonumber \\&v^{'}_{z}(x,y,0)=v_z(x,y,0)=0,~~~ v^{'}_{l}(x,y,0)=v_l(x,y,0), \end{aligned}$$

where *l* can be either *x* or *y*. Note that a perturbative solution to this complementary problem (up to leading orders of *a*/*h*), is presented in Eq. (14). The left-hand side of the Lorentz relation (Eq. 21) vanishes then, we will have:

27 $$\begin{aligned} \nabla \cdot (\Sigma \cdot {\mathbf{u}} _1^{\text {s}}-{\mathbf{T}} _1^{\text {s}}\cdot {\mathbf{v}} )=0. \end{aligned}$$

Integrating this equation over the domain of the lower fluid, will give us:

28 $$\begin{aligned} \int _{A_{M}} {\mathbf{u}} _1^{\text {s}}\cdot \Sigma \cdot {\hat{z}} d^2{\mathbf{x}} - \int _{A_M}{} {\mathbf{v}} \cdot {\mathbf{T}} _1^{\text {s}}\cdot {\hat{z}} d^2{\mathbf{x}} -{\mathbf{V}} \cdot \int _{A_{\text {s}}}{} {\mathbf{T}} _1^{\text {s}}\cdot \hat{\mathbf{n }} d^2{\mathbf{x}} =0. \end{aligned}$$

Repeating a similar procedure over the domain of upper fluid and combining the result with the above relation, we will have:

29 $$\begin{aligned} {\mathbf{V}} \cdot {\mathbf{F}} ^{\text {s}}_1= -\int _{A_{M}} \Delta {\mathbf{u}}_{1}^{\text {s}}\cdot \Sigma '\cdot {\hat{z}} d^2{\mathbf{x}} +\int _{A_{M}} {\mathbf{u}} \cdot \Delta \Sigma \cdot {\hat{z}} d^2{\mathbf{x}} - \int _{A_M}{} {\mathbf{v}} \cdot \Delta {\mathbf{T}} ^{\text {s}}_1\cdot {\hat{z}} d^2{\mathbf{x}} . \end{aligned}$$

As $\hat{\mathbf{n }}$ points into the fluid, $-\int _{A_s}T.\hat{\mathbf{n }}$ is the force exerted from the particle to the fluid. As one can see, the first order hydrodynamic force acting on the particle has a component perpendicular to the wall.

This perpendicular force is quadratic in terms of $U_{\text {p}}$. In Fig. 2b, the first order contribution to the force is plotted in terms of distance from wall and for various values of $\lambda$. This repulsion (from wall) force will increase for larger values of $\lambda$. Expanding this force for small *a*/*h*, the following result can be presented:

30 $$\begin{aligned} &\epsilon {\mathbf{F}} {^s_1}=-\frac{3}{4}Ca\times {6\pi \eta a U_{\text {p}}}\times \frac{a}{h}\times \Gamma _0 ~\hat{z}+{\mathscr {O}}\left( \frac{a}{h}\right) {^3}. \end{aligned}$$

In terms of $U_{\text {p}}$, the above quadratic force can be expressed as: $-(9/2\gamma )\pi a\eta ^2 U_{\text {p}}^2({a}/{h}) \Gamma _0$.

**Figure 6:**
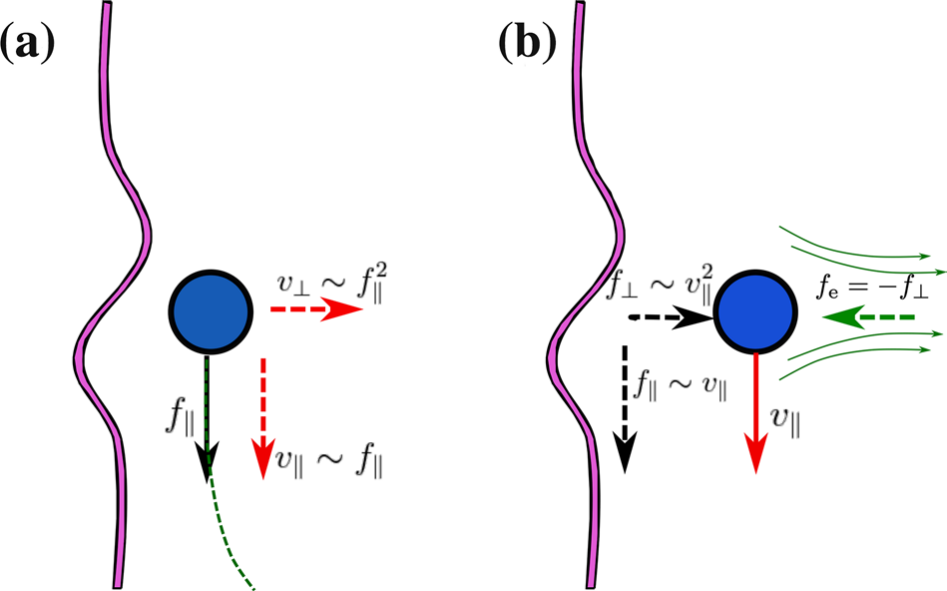
(**a**): A mobility problem where a particle under the action of an external force $f_\parallel$ moves near a soft wall. This particle experiences both parallel and perpendicular motions with corresponding speeds proportional to $f_\parallel$ and $f_{\parallel }^{2}$, respectively. This will eventually form a repelling trajectory (from the wall) for the particle. (**b**): Our resistivity problem where, a particle is constraint to move parallel to a soft wall with a given velocity $v_\parallel$. The hydrodynamic force acting on this particle will have both parallel and perpendicular components proportional to $v_{\parallel }$ and $v_{\parallel }^{2}$, respectively. In this resistivity problem, to keep a fixed distance from the wall, we should apply an external force that is perpendicular to the wall with $f_{\text {e}}=-f_{\perp }$ to the particle. This hydrodynamic pushing force is a result of a repelling (pumping) flow that is back scattered from the wall. Pumping flow that is the nonlinear part of the velocity profile (first order flow) is shown in this figure.

In Fig. 6, we summarize our results and compare it with the results of a mobility problem, presented elsewhere^16^. Part (a) shows the results of a mobility problem where, a particle under the action of external force $f_{\parallel }$ moves near a soft wall. As a result of interface flexibility, the particle will achieve a parallel velocity $v_{\parallel }\propto f_{\parallel }$ and a perpendicular velocity $v_{\perp }\propto f_{\parallel }^2$. The results of our resistivity steady state problem in presented is Fig. 6b, where for a parallel velocity $v_{\parallel }$, parallel force $f_{\parallel }\propto v_{\parallel }$ and perpendicular force $f_{\perp }\propto v_{\parallel }^2$ will act on the particle. The direction of first order flow that is quadratic in terms of $U_{\text {p}}$ is shown in this figure. This pumping flow points toward $-\hat{z}$ direction.

## Average pumping flow

After finding the steady state solutions to the problem of a moving sphere near a wall, we can use Eq. (3), and find the time dependent flow pattern as:

$$\begin{aligned} {\mathbf{u}}(x,y,z,t)={\mathbf{u}}^{\text {s}}(x-x_{\text {p}}(t),y,z). \end{aligned}$$

Due to the quasi-steady approximation that we have used, the time dependence in the results is only due to the time evolution of $x_{\text {p}}(t)$ or subsequently $U_{\text {p}}(t)$. For our sinusoidal time evolution of the back and forth motion, the zeroth order result for the velocity profile and the hydrodynamics forces acting on the sphere, those are proportional linearly to the velocity, will vanish on average. In zeroth order approximation the membrane remains flat. The first order corrections take into account the deformation of the membrane and these corrections are quadratic in particle’s velocity. Such terms will have finite contribution when we average over time. Denoting by $T=2\pi /\omega$, the period of back and forth motion, the average velocity profile in a given point can be calculated as:

31 $$\begin{aligned} \langle {\mathbf{u }}({\mathbf{x}} )\rangle =\frac{1}{T}\int _{T}{} {\mathbf{u}} ({\mathbf{x}} ,t) dt=\frac{1}{T}\int _{T}({\mathbf{u}} _0({\mathbf{x}} ,t)+\epsilon {\mathbf{u}} _1({\mathbf{x}} ,t)) dt=\langle \epsilon {\mathbf{u }}_1({\mathbf{x}} )\rangle , \end{aligned}$$

as we expected, only the first order correction contributes to the average velocity.

**Figure 7:**
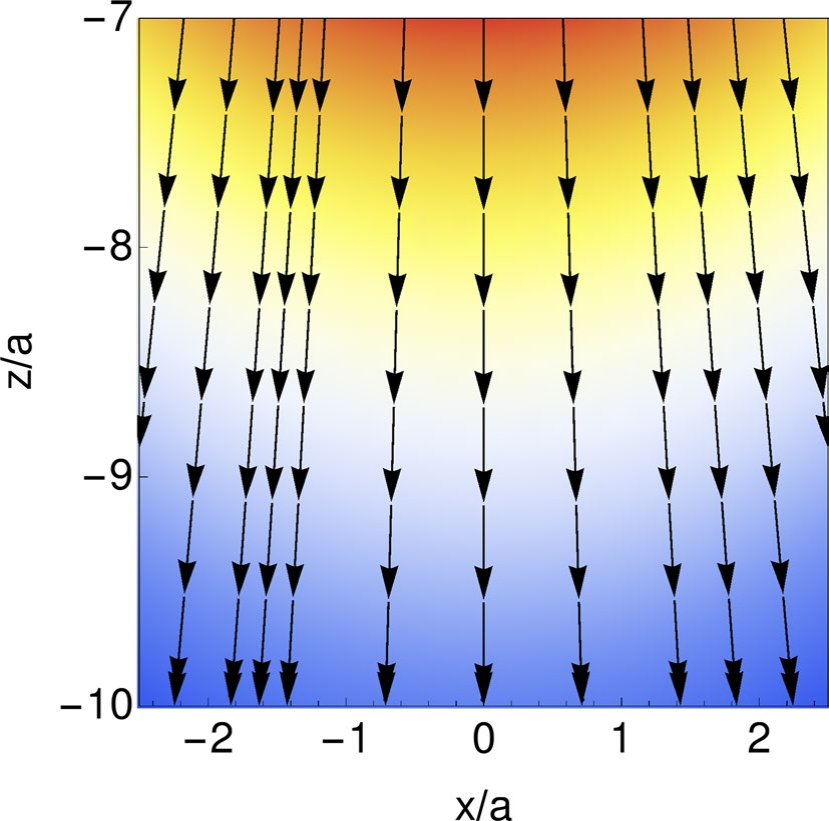
The first order streamlines are shown for $\lambda =0$, ${\text {Ca}}=0.1$ and $h/a=5$.

Figure 7 shows the averaged streamlines far below the position of the moving particle. As one can distinguish two important features of the streamlines include its right-left symmetry and the direction of averaged flow. Right-left symmetry is a fact that we expected from the beginning. Interface is isotropic and back and forth motion of the particle is symmetric as well. There is no reason to break the left-right symmetry. But, the up-down symmetry is broken, evidently from the geometrical constraints.

Reflected in Fig. 7, the direction of the averaged flow for $\lambda =0$ is perpendicular to the interface in a way that it tends to repel the particle from interface. To have an intuition about the mechanism behind this repulsing flow, one can see from Fig. 4, the boundary condition for the first order flow. Actually this is the back flow corresponding to the zeroth order problem that needs to satisfy the boundary conditions correctly. As one can see, the inlet flow is concentrated on top of the particle and it points toward the particle. Propagation of this boundary flow, will repel the particle from boundary. Figure 6b also helps to obtain feeling on the pumping flow. As one can see from this figure, independent of the direction of parallel motion (up or down in the figure), a nonlinear contribution to the perpendicular force will arise that is always repelling. The pumping flow is solely due to this repelling force.

**Figure 8:**
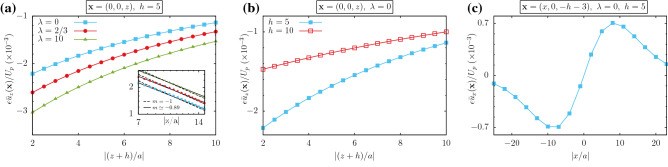
(**a**) Averaged pumping velocity in terms of the distance from the wall for various values of $\lambda$. Absolute value of the velocity will increase for larger values of $\lambda$. Inset shows power law fittings in logarithmic scale. Velocity scales as $z^{m}$ , with $m=-0.89$ that is very near to the scaling behavior $m=-1$ resulted from dimensional analysis. (**b**) The pumping velocity for different values of *h*. (**c**) Parallel component of the flow just below the particle, has left-right symmetry. Including ${\text {Ca}}=0.1$, other parameters are mentioned in the figures.

Variation of the averaged pumping velocity is studied in Fig. 8. The pumping velocity is plotted in Fig. 8a in terms of the separation from wall. Increasing $\lambda$, will increase the strength of pumping flow. Fig. 8b shows the role of micro-particle position in the pumping flow. As one can see, micro-particles that are near to the wall can produce more stronger perpendicular flow. The parallel component of the flow just below the particle is plotted in part (c). As expected from the beginning the left-right symmetry is reflected in this figure.

## Dimensional analysis

Here we use simple dimensional arguments to obtain the order of magnitude for the fluid velocity created by a moving micro-particle behind the interface. Let us assume that the particle velocity is given by ${\mathbf{U}}(t)=U_{\text {p}}\cos (\omega t) {\hat{x}}$ and it is located in a fixed position in a distance *h*, below the interface. This small sphere can be replaced by a time dependent point force given by ${\mathbf{f}}(t)=6\pi \eta a U_{\text {p}}\cos (\omega t) {\hat{x}}$. We want to estimate the fluid velocity in a position just below the point force, with a distance $H\gg h$ from the wall. As a result of right-left symmetry, the flow averaged over time, will not have any component parallel to the membrane. Perpendicular component of the flow needs more inspection.

To obtain the flow velocity, we follow the same steps as we have defined in the perturbation analysis. In order to estimate the quantities, we can distinguish two different limiting regimes. First regime corresponds to the case where, the gravitational and capillary effects have the same order of magnitudes. In this case $\alpha \sim \beta$ and capillary length $\ell _{\text {c}}$, is a relevant length scale in the system. Second regime corresponds to the case where, gravitational effects are negligible ($\alpha \sim 0$). In this regime, the capillary number ${\text {Ca}}=\beta ^{-1}$, is relevant dimensionless number that takes into account the deformability of the interface.

We begin by the first regime and estimate the instantaneous deformation of the membrane. We denote by $\Delta$ and $\delta$, the lateral and normal deformations of the interface, respectively. Looking to the left hand side of Eq. (11), we see that the capillary length is the natural length scale for the lateral deformation, $\Delta \sim \ell _{\text {c}}$. Using the same equation and Eq. (16), and replacing by $\Delta$, the variable *r* that appears at the right hand side, we see that:

32 $$\begin{aligned} \delta \sim \frac{f(t)}{g\Delta \rho }\times \frac{\ell _{\text {c}}}{h^3},~~~~~~{\text {for}}~~h\gg \ell _{\text {c}}. \end{aligned}$$

Our previous perturbative formalism, showed that the first order velocity field is subjected to inlet velocity field given on the undeformed membrane. The inlet profile on the membrane can be estimated from Eq. (13), with a typical term given by $u_{1z}(z=0)\sim u_0\partial _x\xi _1$:

33 $$\begin{aligned} \epsilon u_{1z}(z=0)\sim \frac{f}{6\pi \eta a}\times \frac{\delta }{\Delta }. \end{aligned}$$

To calculate the first order velocity field, we use a typical term from Eq. (23) (Lorentz integral), as:

34 $$\begin{aligned} {\mathbf{u}} ^{\text {}}_1({\mathbf{x}} _0)\cdot {\mathbf{f}} _0\sim \int _{A_{F}} {u}^{\text {}}_{1z}\Sigma _{zz} d^2{\mathbf{x}} , \end{aligned}$$

where $f_0$ is a point force in the observation point and the stress tensor corresponding to this point force scales as $\Sigma \sim (f_0/H^2)$. Estimating the domain of integration by $\Delta$, this will give us the following average velocity:

35 $$\begin{aligned} \langle \epsilon u_{1z}\rangle \sim \frac{\langle f(t)^2\rangle }{\eta \gamma a^2}~\left( \frac{a}{h}\right) ~\left( \frac{\ell _{\text {c}}}{h}\right) ^2~\left( \frac{\ell _{\text {c}}}{H}\right) ^2\times \psi \left( \frac{\ell _{\text {c}}}{h},\frac{h}{H},\frac{a}{h}\right) , \end{aligned}$$

where $\psi$ is a dimensionless function with $\psi (0,0,0)\sim 1$. As it is reflected from above relation, the velocity is nonlinear in terms of *f* and it averages to a finite value with $\langle f(t)^2\rangle \ne 0$.

For the second regime with $\alpha =0$, we can see that *h* is a natural length scale that limits the lateral deformation so that $\Delta \sim h$. Using Eq. (11), we see that $\delta \sim {\text {Ca}}\times a$. Now following the same procedure as we used for the first regime, we will arrive at the following result:

36 $$\begin{aligned} \langle \epsilon u_{1z}\rangle \sim \frac{\langle f(t)^2\rangle }{\eta \gamma a^2}~\left( \frac{a}{h}\right) ~\left( \frac{a}{H}\right) \times \phi \left( \frac{h}{H},\frac{a}{h}\right) , \end{aligned}$$

where $\phi$ is a dimensionless function with $\phi (0,0)\sim 1$. Inset in Fig. 8a, shows that the scaling with *H* works good.

## Discussion

In this article we show how a simple one dimensional periodic motion of a colloidal particle near a soft wall can break the symmetry and produce an averaged flow in a preferred direction perpendicular to the wall. The hydrodynamic problem that we have solved, is a resistivity problem where the components (parallel and perpendicular) of the particle’s velocity are given. It should be noted that in current case where the dynamics is highly nonlinear, the mobility and resistivity problems are not equivalent. In mobility problem, instead of velocity, the force components acting on the particle are given^23^.

We use a perturbative method to analytically solve the problem and study the pumping velocity in terms of some physical parameters of the system. In addition to small deformation assumption and large distance ($h\gg a$) approximation, we have assumed that the amplitude of the back and forth motion is also small. Another important simplification that we have used, is associated with neglecting the rotational motion of the colloidal particle. All of our results correspond to large capillary length with small capillary number.

As it is mentioned in the article, the deformability of the boundary is the main element that helps to produce flow through a setup that can be easily accessible in experiments. As a source of flexibility, we address the surface tension in an interface but one can imagine a complicated surface with different kind of elastic modulus. Bending flexibility is another common properties that most surfaces have. A very simple dimensional analysis can shed light on the physical behavior of systems that have a single bending rigidity denoted by $\kappa$. To take into account the bending rigidity, we should modify Eq. (11) by replacing $\gamma \rightarrow \left( \gamma -\kappa \nabla ^2\right)$. In this case another length scale denoted by $\ell _{\text {B}}=\sqrt{\kappa /\gamma }$ enters into the dynamics of system. As a result of this bending nonlinearity, a normal force and subsequently, pumping flow in the resistivity problem will be expectable. In this case, a new dimensionless number $\ell _{\text {B}}/\ell _{\text {c}}$ will enter into the non-dimensional functions appeared in Eqs. (35) and (36). The corresponding normal force is studied for a mobility problem in recent works^16, 17^.

It should be mentioned that, the micro-particle can move perpendicular to the wall instead of a parallel motion. Based on symmetry considerations, for a perpendicular back and forth motion and on average, nothing expected to see in the parallel direction. But we expect to obtain a nonlinear contribution to the perpendicular force that can mediate an average flow in that direction.
